# Emergency surgery for hemobilia due to hepatic artery pseudoaneurysm rupture complicated by Mirizzi syndrome type II: a case report

**DOI:** 10.1186/s12893-021-01314-z

**Published:** 2021-08-05

**Authors:** Yutaka Shishido, Koji Fujimoto, Yasumichi Yano, Eisei Mitsuoka, Takashi Komatsubara, Seiji Shio, Masayuki Ishii, Hiroshi Higashiyama

**Affiliations:** 1 Department of Gastrointestinal Surgery, Shinko Hospital, 1-4-47, Wakinohama-cho, Chuo-ku, Kobe, Hyogo 6510072 Japan; 2 Department of Gastroenterology, Shinko Hospital, 1-4-47, Wakinohama-cho, Chuo-ku, Kobe, Hyogo 6510072 Japan

**Keywords:** Hemobilia, Pseudoaneurysm, Hepatic artery, Mirizzi syndrome, Surgery, Case report

## Abstract

**Background:**

Hemobilia refers to bleeding into the biliary tract. Hepatic artery pseudoaneurysm (HAP) rupture is an uncommon cause of hemobilia, and cases of HAP associated with Mirizzi syndrome are extremely rare. Although transarterial embolization is recommended as the first-line treatment for hemobilia, surgery is sometimes required.

**Case presentation:**

A 76-year-old woman was referred to our hospital with epigastric pain. She was febrile and had conjunctival icterus and epigastric tenderness. Laboratory tests revealed abnormal white blood cell count and liver function. An abdominal computed tomography (CT) revealed multiple calculi in the gallbladder, an incarcerated calculus in the cystic duct, and a slightly dilated common hepatic duct. Based on examination findings, she was diagnosed with Mirizzi syndrome type I, complicated by cholangitis. Intravenous antibiotics were administered, and we performed endoscopic retrograde cholangiopancreatography (ERCP) to place a drainage tube. The fever persisted; therefore, contrast-enhanced CT (CECT) was performed. This revealed portal vein thrombosis and hepatic abscesses; therefore, heparin infusion was administered. The following day, she complained of melena, and laboratory tests showed that she was anemic. ERCP was performed to change the drainage tube in the bile duct; however, bleeding from the papilla of Vater was observed. CECT demonstrated a right HAP with high-density fluid in the gallbladder and gallbladder perforation. Finally, she was diagnosed with hemobilia caused by HAP rupture, and emergency surgery was performed to secure hemostasis and control the infection. During laparotomy, we found that a right HAP had ruptured into the gallbladder. The gallbladder made a cholecystobiliary fistula, which indicated Mirizzi syndrome type II. Although we tried to repair the right hepatic artery, we later ligated it due to arterial wall vulnerability. Then, we performed subtotal cholecystectomy and inserted a T-tube into the common bile duct. There were no postoperative complications except for minor leakage from the T-tube insertion site. The patient was discharged after a total hospital stay of 7 weeks.

**Conclusions:**

We experienced an extremely rare case of emergency definitive surgery for hemobilia due to HAP rupture complicated by Mirizzi syndrome type II. Surgery might be indicated when controlling underlying infections was required.

## Text

### Background

Hemobilia refers to bleeding into the biliary tract, which could be life-threatening [1]. The major causes of hemobilia are iatrogenic or accidental trauma and malignancy. Hemobilia remains an uncommon cause of gastrointestinal bleeding; however, its incidence has gradually increased because of the development of hepatopancreatobiliary interventions [2, 3]. Hepatic artery pseudoaneurysm (HAP) rupture is an unusual cause of hemobilia. There are many etiologies of HAP formation [4]; however, HAP associated with Mirizzi syndrome is extremely rare. Mirizzi syndrome is a chronic complication of gallstone disease and is classified into 3 types, based on the presence of a cholecystobiliary fistula and cholecystoenteric fistula [5]. Although transarterial embolization (TAE) is recommended as the first-line treatment for hemobilia caused by HAP rupture, surgery is indicated for other reasons or if TAE fails [3]. Herein, we report an extremely rare case of emergency definitive surgery for hemobilia due to HAP rupture that was complicated by Mirizzi syndrome type II.

## Case presentation

**Fig. 1:**
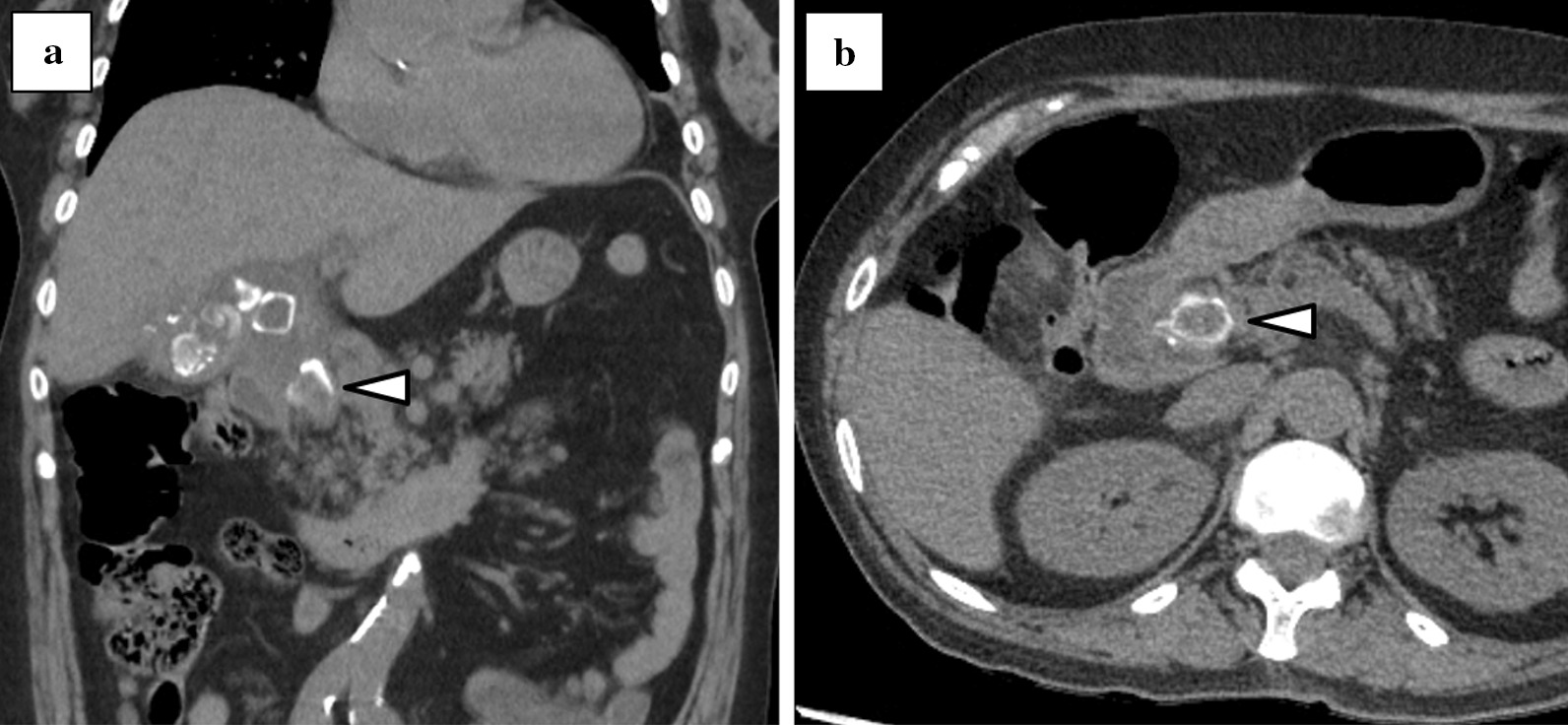
Abdominal computed tomography at admission (**a**) Multiple calculi in the gallbladder and an incarcerated calculus in the cystic duct (triangle) (**b**) An incarcerated calculus in the cystic duct (triangle)

**Fig. 2:**
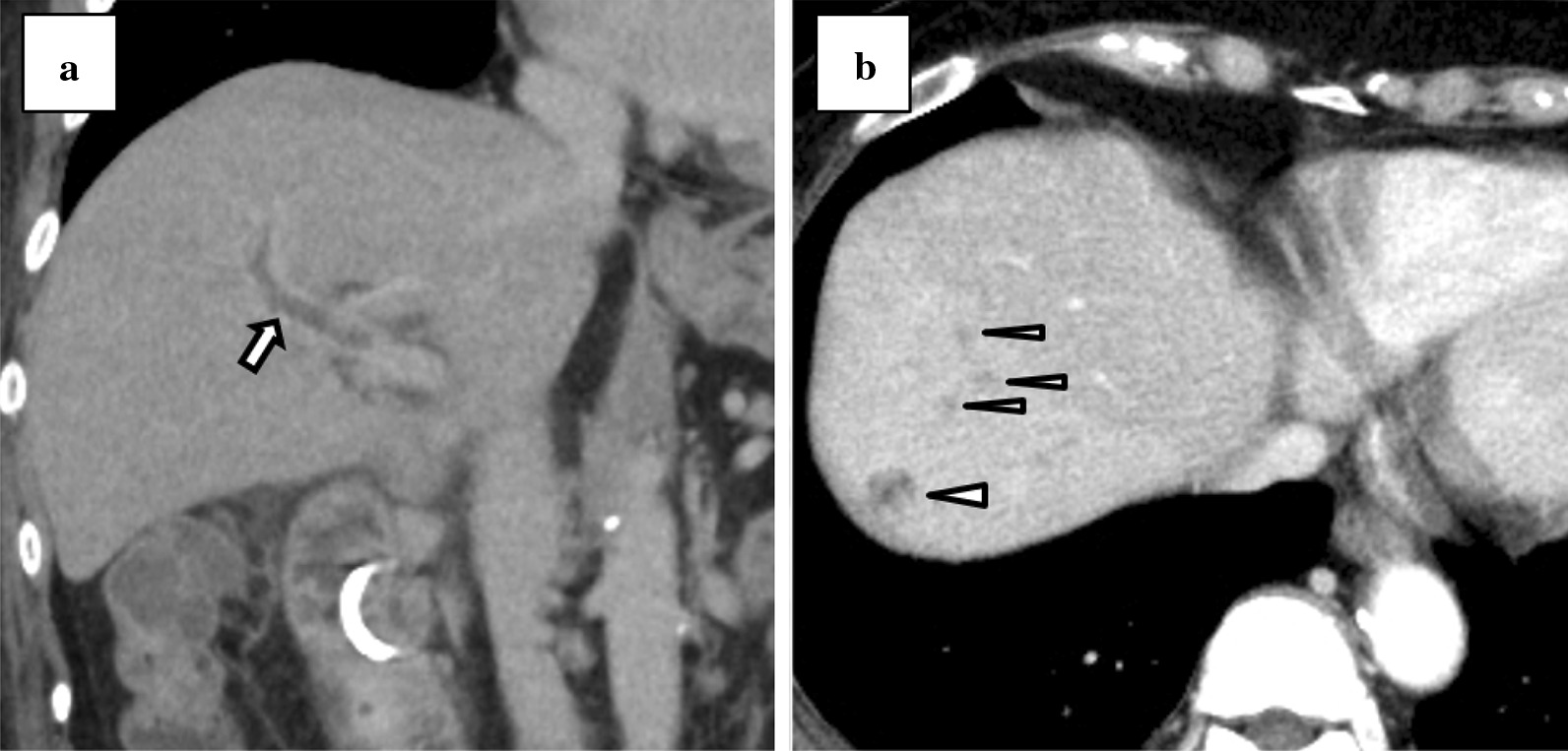
Contrast-enhanced computed tomography on day 7 after admission (**a**) Portal vein thrombosis (arrow) (**b**) Multiple hepatic abscesses (triangles)

**Fig. 3:**
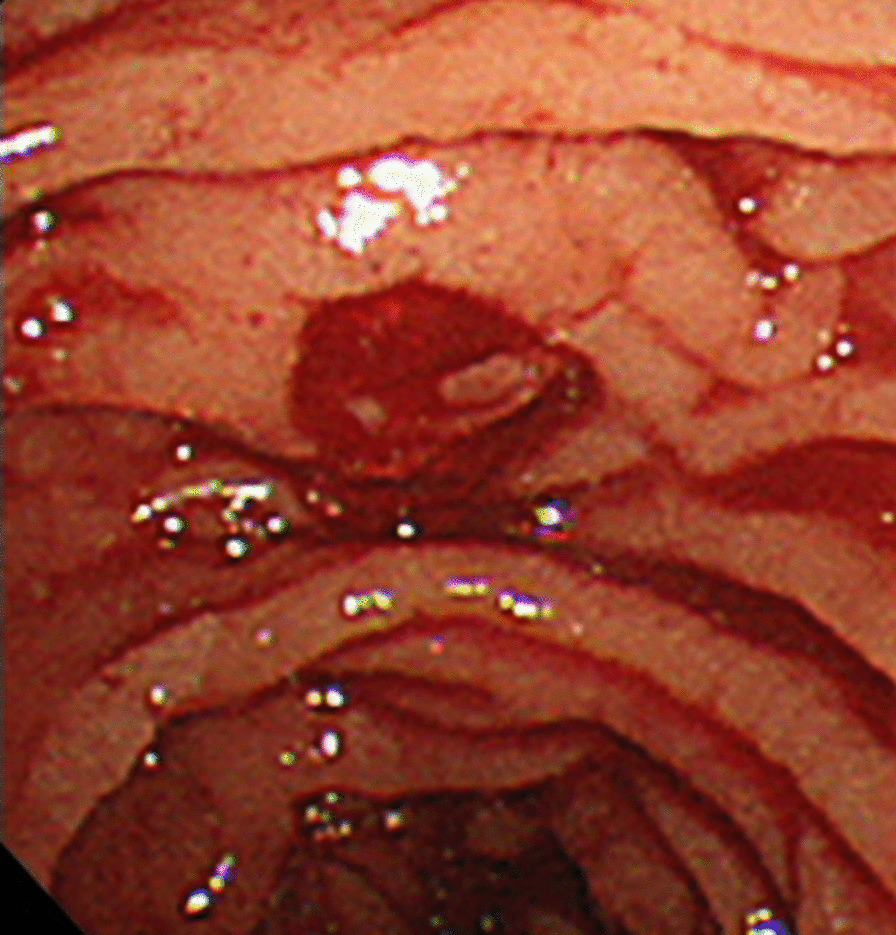
Endoscopic retrograde cholangiopancreatography reveals fresh blood oozing from the papilla of Vater

**Fig. 4:**
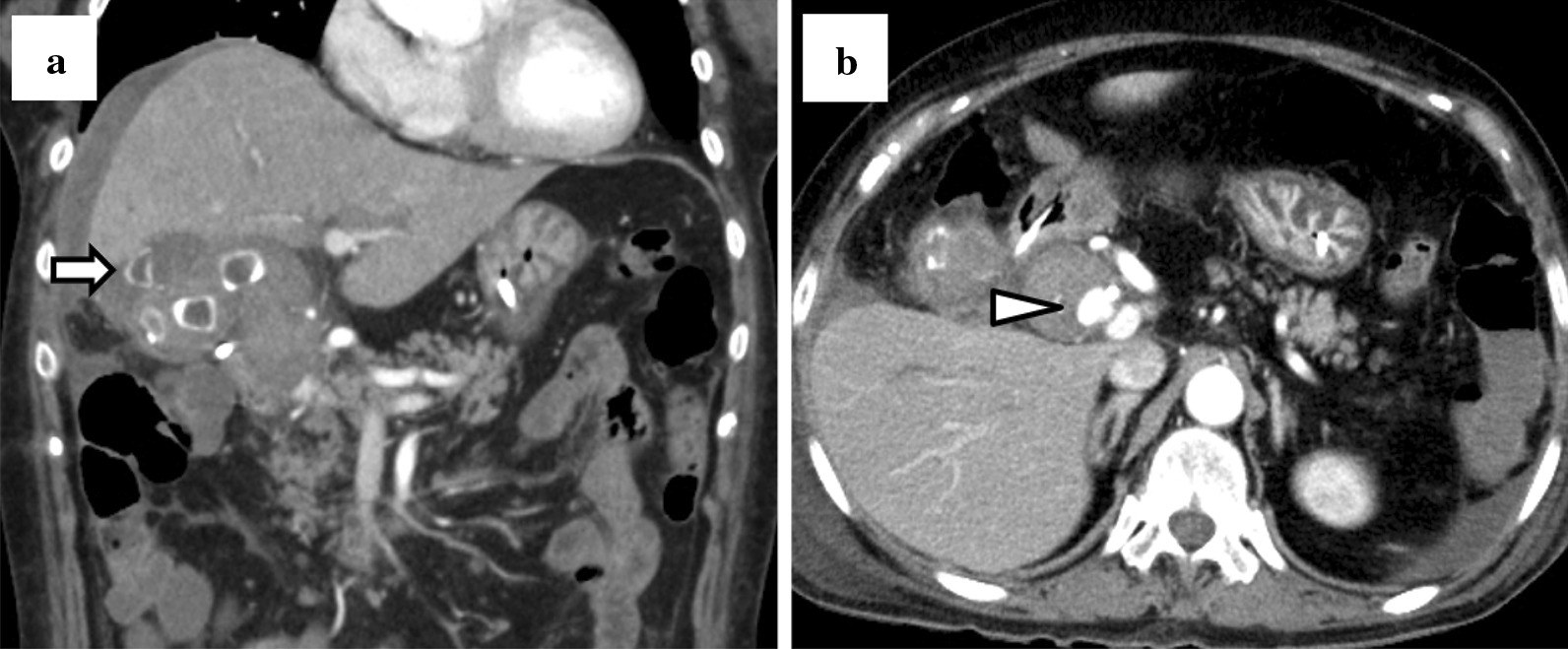
Preoperative abdominal computed tomography (**a**) Perihepatic ascites, and a calculus inside and outside the gallbladder (arrow) (**b**) A contrast-filled sac bulging from the right hepatic artery (triangle)

A 76-year-old woman was referred to our hospital with a 1-month history of postprandial epigastric pain. She had a medical history of hypertension and hyperlipidemia. On examination, she had stable vital signs except for a fever of 37.9 degrees Celsius. Physical examination revealed conjunctival icterus and mild epigastric tenderness. Laboratory tests indicated a white blood cell (WBC) count of 20,400/µL, hemoglobin (Hb) concentration of 10.3 g/dL, and platelet count of 2.88 × 10^5^ /µL. Her liver function tests revealed total bilirubin of 3.3 mg/dL, aspartate aminotransferase of 366 IU/L, alanine aminotransferase of 399 IU/L, alkaline phosphatase of 1752 IU/L, and γ-glutamyltransferase of 1257 IU/L. Her other laboratory results were within normal limits. An abdominal computed tomography (CT) revealed multiple calculi in the gallbladder, an incarcerated calculus in the cystic duct, and a slightly dilated common hepatic duct (Fig. 1a–b). Based on the findings, she was diagnosed with Mirizzi syndrome type I complicated with obstructive cholangitis. At admission, we performed emergency endoscopic retrograde cholangiopancreatography (ERCP) and inserted an endoscopic retrograde biliary drainage (ERBD) tube. Her symptoms and liver function tests, except for the low-grade fever, improved after administration of intravenous ampicillin-sulbactam. On day 7 of admission, contrast-enhanced CT (CECT) was performed to identify the cause of the persistent fever, and this revealed thrombosis at the right anterior branch of the portal vein and multiple hepatic abscesses (Fig. 2a–b). Therefore, heparin infusion was administered and we planned to change the ERBD tube to an endoscopic nasobiliary drainage (ENBD) tube because we assumed that the ERBD tube compressed the portal vein and caused the formation of thrombi. The following day, she complained of melena, and her Hb concentration was 7.6 g/dL. We stopped the heparin infusion and performed esophagogastroduodenoscopy, which revealed multiple gastroduodenal ulcers. The ulcers were not bleeding and hence were prophylactically treated with endoscopic clips. The next morning, her Hb level decreased to 6.1 g/dL. As she was already scheduled for a change of an ERBD tube to an ENBD tube, she was transfused 4 units of packed red blood cells and ERCP was performed. ERCP revealed fresh blood oozing from the papilla of Vater, which was suggestive of hemobilia (Fig. 3). After the procedure, there was no evidence of ongoing bleeding from the ENBD tube, and her Hb level increased to 9.6 g/dL owing to the transfusion. However, 2 days later, she had a high-grade fever, and laboratory tests revealed a WBC count of 26,100/µL and Hb of 6.7 g/dL. CECT revealed perihepatic ascites, high-density fluid within the gallbladder, calculi outside the gallbladder, and a contrast-filled sac bulging from the right hepatic artery (RHA) (Fig. 4a–b). Finally, she was diagnosed with hemobilia caused by a hepatic artery pseudoaneurysm (HAP) that ruptured into the gallbladder, along with gallbladder perforation. We decided to perform emergency surgery to control hemorrhage and generalized peritonitis associated with gallbladder perforation.

**Fig. 5:**
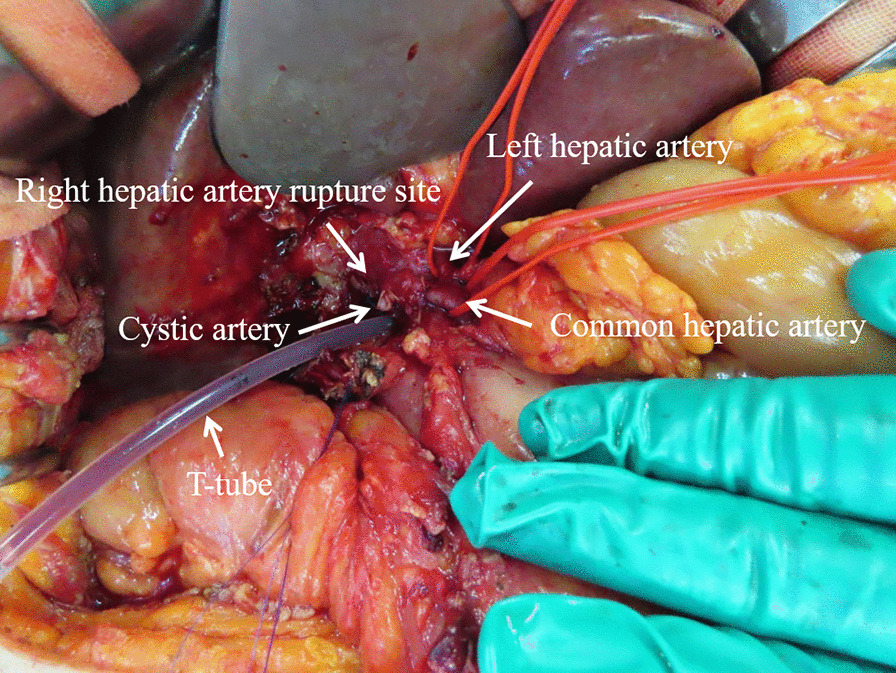
Intraoperative abdominal image after right hepatic artery ligation, subtotal cholecystectomy, and T-tube insertion

During laparotomy, we noticed blood-stained ascitic fluid and blood clots around the liver, and that the omentum had adhered to the liver and the gallbladder. There were also strong pericholecystic adhesions. We secured the common hepatic artery and the RHA in preparation for the uncontrolled intraoperative bleeding. After clamping the RHA, we carefully separated the gallbladder from the liver bed. The gallbladder was perforated and filled with multiple gallstones and blood clots, which we removed. Subsequently, we identified active bleeding from the fistula between the gallbladder and ruptured HAP. We initially tried to repair the RHA; however, the arterial wall was very vulnerable. Therefore, we ligated the RHA in view of the risk of postoperative bleeding. After securing hemostasis, we performed subtotal cholecystectomy because of the presence of intense inflammation around the gallbladder neck. There was a fistula between the gallbladder and the common bile duct, which indicated Mirizzi syndrome type II. We inserted a T-tube into the common bile duct (Fig. 5), and intraoperative cholangiography illustrated no leakage of contrast media or strictures in the common bile duct. The ENBD tube was left in place.

Postoperatively, she was kept nil per oral and administered parenteral crystalloids. Intravenous piperacillin-tazobactam was administered for 2 weeks and stopped after the improvement of inflammation with blood tests was confirmed. On postoperative day 3, cholangiography demonstrated mild leakage from the insertion site of the T-tube, which was managed conservatively. The ENBD tube was removed because it got obstructed and adequate drainage was achieved with the T-tube. She also commenced oral fluid intake. On postoperative day 8, CECT illustrated that the HAP had disappeared, the hepatic abscesses had reduced, and there were no signs of hepatic ischemia. On postoperative day 29, cholangiography revealed no leakage, and the T-tube was removed. Although the RHA was ligated, there were no postoperative necrotic complications, such as liver abscesses in the anterior region of the right hepatic lobe. Subsequently, she commenced oral feeds and was discharged after a total hospital stay of 7 weeks. At 6 months after the surgery, there were no abdominal symptoms, and follow-up blood tests and abdominal ultrasound did not reveal any abnormalities.

## Discussion and conclusion

Hemobilia refers to bleeding into the biliary tract that occurs when an abnormal fistula is formed between the bile duct and a blood vessel of splanchnic circulation [1]. The bleeding is often minor, but could be life-threatening and may require urgent interventions. There are many possible causes of hemobilia, including iatrogenic and accidental trauma, malignancy, gallstones, infection, and vascular diseases [2, 3]. Although hemobilia remains an uncommon cause of gastrointestinal bleeding, its incidence has gradually increased due to the development of interventional hepatopancreatobiliary procedures [2, 3]. In this case, a HAP complicated by Mirizzi syndrome ruptured into the gallbladder and caused hemobilia. Mirizzi syndrome is a chronic complication of symptomatic gallstone disease. An impacted gallstone at the infundibulum of the gallbladder causes external obstruction of the bile duct (Mirizzi syndrome type I). This eventually erodes into the bile duct and evolves into a cholecystobiliary fistula (Mirizzi syndrome type II) [5]. To date, there are only two reported cases where HAP was associated with Mirizzi syndrome, and these cases were treated with a combination of TAE and surgery [6, 7]. Lin et al. performed cholecystectomy and RHA ligation for a HAP rupture complicated by Mirizzi syndrome type I [6]. On the other hand, Anderson and coworkers conducted cholecystectomy and embolization of the HAP in a patient with HAP rupture caused by Mirizzi syndrome type II [7]. In this study, we report a case of emergency subtotal cholecystectomy and RHA ligation for hemobilia due to HAP rupture complicated by Mirizzi syndrome type II.

HAP is mostly caused by direct vascular injury due to accidental or iatrogenic hepatobiliary trauma [4]. Bile is known to cause endothelial damage to blood vessels, and vascular irritation due to bile leakage after surgical interventions could lead to the formation of pseudoaneurysms [8]. In patients who undergo sphincterotomy, reflux of duodenal chime into the biliary tract might cause continuous low-grade injury to the liver parenchyma, which leads to the formation of HAP [9]. Pancreatitis could be attributed to the formation of HAP due to pancreatic enzymes that cause autodigestion of the elastic fiber skeleton supporting the arterial wall [4]. Although the exact mechanism remains unclear, infectious or inflammatory conditions could also result in the formation of HPA [10]. In our case, the patient had obstructive cholangitis at admission, which was later complicated by cholecystitis, multiple hepatic abscesses, and eventually advanced to gallbladder perforation. This severe inflammatory process might have infiltrated connective tissues within the hepatoduodenal ligaments, partially eroded the vascular wall, and contributed to the formation of HAP.

Due to its rarity, there is no unanimously agreed treatment for hemobilia caused by HPA rupture. Although TAE is recommended as the first-line intervention for hemobilia, surgery is indicated for other reasons or if TAE fails [3]. In the present case, we performed emergency surgery as a definitive treatment to achieve hemostasis and to control infection and peritonitis caused by gallbladder perforation. Preoperatively, the infection was getting worse, even though broad-spectrum antibiotics were being administered and an ENBD tube had been placed. Therefore, we concluded that radical treatment for Mirizzi syndrome should be performed as early as possible. Moreover, she had hepatic abscesses and this was a relative contraindication for TAE [11]. In terms of hepatic blood flow, our patient had portal vein thrombosis that compromised collateral blood flow from the portal vein and predisposed the patient to liver ischemia due to arterial embolization [12]. Furthermore, our radiologists were concerned about the risk of embolic materials accidentally migrating into the left hepatic artery, because the location of the right HPA was close to the bifurcation of the proper hepatic artery. Hence, we tried to repair the RHA surgically and preserve liver blood flow; however, we performed arterial ligation due to the vulnerability of the arterial wall. We also considered reconstructing the RHA using other arteries, such as the gastroduodenal artery, the splenic artery, or the first jejunal artery, as a graft; however, due to the severe pericholecystic inflammation, the damage of the arterial wall extended over the entire length of the RHA and we could not identify the optimal anastomotic site distal to the bifurcation of the proper hepatic artery. Fortunately, ligation of the RHA did not cause postoperative necrotic complications, such as liver abscess in the anterior region of the right hepatic lobe. Although our patient was hemodynamically stable after receiving blood transfusions and tolerated surgery under general anesthesia, TAE might be indicated as a temporary treatment if the patient was hemodynamically unstable.

In conclusion, we experienced an extremely rare case of hemobilia due to HAP rupture complicated by Mirizzi syndrome type II managed successfully by emergency definitive surgery. Surgery might be indicated as a first-line treatment for hemobilia due to HAP rupture when control of underlying infections is required.

## Data Availability

The datasets used and/or analysed during the current study are available from the corresponding author on reasonable request.

## Abbreviations

HAP: Hepatic artery pseudoaneurysm
CT: Computed tomography
ERCP: Endoscopic retrograde cholangiopancreatography
CECT: Contrast-enhanced CT
TAE: Transarterial embolization
WBC: White blood cell
Hb: Hemoglobin
ERBD: Endoscopic retrograde biliary drainage
ENBD: Endoscopic nasobiliary drainage
RHA: Right hepatic artery
